# Place de l´échographie endovaginale dans l´exploration de l´infertilité d´origine endométriale

**DOI:** 10.11604/pamj.2020.37.92.22375

**Published:** 2020-09-25

**Authors:** Sanae Stimou, Hafsa Taheri, Hanane Saadi, Ahmed Mimouni

**Affiliations:** 1 Service de Gynécologie Obstétrique au Centre Hospitalier Universitaire Mohammed VI d´Oujda, Oujda, Maroc

**Keywords:** Echographie endovaginale, endomètre, infertilité, hypofertilité, Transvaginal ultrasound, endometrium, infertility

## Abstract

Il est important d´explorer la cavité utérine dans le bilan d´une infertilité, car de nombreuses lésions intra-utérines peuvent être retrouvées. L´échographie endovaginale (EEV) est un examen de première intention dans le bilan d´une infertilité féminine. Elle permet une évaluation de la cavité utérine à la recherche d´anomalie responsable du trouble de la fertilité et aussi de mettre en évidence des lésions pouvant entraîner des échecs de transfert ou d´implantation. Il s´agit d´un examen facilement réalisable, et reproductible. Notre objectif est de détailler les lésions endométriale détectable par EEV pour préciser la place de l´EEV dans le bilan d´infertilité.

## Abstract (en)

The assessment of the uterine cavity in patients with infertility is essential as many intrauterine lesions can be detected. Transvaginal ultrasound (TVE) is a front-line testing for female infertility. It allows assessment of the uterine cavity for detection of abnormalities responsible for fertility disorder as well as of lesions that may lead to transfer or implantation failures. It is an easily perfomable and reproducible exam. The purpose of our study is to describe endometrial lesions detectable on TVE in order to highlight the role of TVE in the evaluation of infertility.

## Introduction

L´infertilité occupe une place importante dans les consultations gynécologiques, l´EEV est un examen important dans le bilan d´une infertilité. Il s´agit d´un examen de première intention dans le bilan d´une infertilité. Les infertilités d´origine exclusivement utérine représentent 2 à 3% des infertilités, cependant, les lésions intra-utérines sont beaucoup plus fréquentes chez les femmes infertiles (40-50%). Ces lésions peuvent interférer avec la fertilité spontanée ou retentir sur les résultats de l´assistance médicale à la procréation (AMP).

Une infertilité de cause endométriale peut être recherchée par de nombreuses techniques: échographie, hystérosalpingographie, IRM, hystéroscopie et biopsie endométriale. L´échographie, spécialement par voie endovaginale, est la première étape dans l´étude de la cavité utérine. Cet examen est bien toléré, il produit une imagerie de haute résolution et il est facilement réalisable, accessible et disponible. L´hystérosonographie (HSO) avec contraste par instillation de sérum physiologique dans la cavité utérine avant l´échographie endovaginale améliore cette imagerie. Notre objectif est de montrer le rôle de cet outil d´imagerie accessible et disponible dans le bilan de l´infertilité féminine et en détaillant les lésions échographiques endométriales afin de mieux cerner la place de l´EEV dans le bilan des hypofertilités et infertilités féminines.

## Séminaire

Selon l´OMS, l´infertilité est l'absence de conception après au moins 12 mois de rapports sexuels non protégés [1]. On distingue 2 catégories d´infertilité: primaire et secondaire. L´infertilité primaire est définie par l´absence de toute grossesse antérieure, alors que dans l´infertilité secondaire le couple a eu une grossesse antérieure. Cette différenciation est importante en raison du meilleur pronostic de l´infertilité secondaire. Cependant, le bilan diagnostique des deux types d´infertilité est identique [2]. L´évaluation de l´infertilité vise à diagnostiquer la cause et à poser le pronostic de la fertilité future. Le bilan doit être initié après un an de tentatives infructueuses de conception, ou plus tôt si une pathologie est suspectée chez l´un des partenaires [3].

L´épaisseur de l´endomètre est mesurée sur une coupe sagittale médiane de l´utérus. L´échogénicité de l´endomètre est déterminée en la comparant à celle du myomètre, l´endomètre ayant un aspect hyperéchogène, isoéchogène ou hypoéchogène par rapport au myomètre en fonction de la date du cycle. La corrélation entre l'épaisseur endométriale et son aspect par rapport à la phase du cycle menstruel est à la base de la surveillance et l'adaptation des traitements de stimulation. Le changement lié à la sensibilité aux hormones se traduit par des modifications de son épaisseur, de son volume et son échogénicité. En début du cycle menstruel, au moment des règles, l´épaisseur de l´endomètre est nulle à très faible (< 2 mm). La cavité utérine est souvent visible, son aspect est hypoéchogène, hétérogène avec parfois des zones hyperéchogènes, correspondant à des caillots et débris de muqueuse abrasée en cours d´évacuation.

En début de phase folliculaire, l´endomètre régénératif se présente sous l´aspect de deux couches hypoéchogènes par rapport au myomètre, souvent confondues en une seule couche, la cavité utérine n´étant pas toujours visible. Ces deux couches vont progressivement s´épaissir et leur séparation centrale qui correspond à l´accolement des deux feuillets superficiels de l´endomètre va devenir de plus en plus nette, se traduisant par une ligne fine plus échogène. Les limites entre l´endomètre et le myomètre sont progressivement de mieux en mieux identifiables et se matérialisent par une ligne légèrement plus échogène. L´endomètre idéal est en triple ligne avec une ligne hyperéchogène centrale entourée de deux couches hypo-échogènes « en grain de café ». Un aspect hyperéchogène est de mauvais pronostic.

L´endomètre augmente progressivement de volume, pour parvenir à une épaisseur entre 7 et 9 mm le jour du pic de LH (*luteinizing hormone*). Une épaisseur inférieure à 7 mm est corrélée à une diminution de chance de grossesse. En phase périovulatoire, l´endomètre se modifie, s´épaissit, mesurant entre 10 et 12 mm. Son aspect devient caractéristique, en cible ou en anneau périovulatoire (*ring sign*) constitué d´une triple ligne (on parle également d´aspect trifolié). La ligne centrale qui correspond à la cavité utérine virtuelle reste fine mais devient très nette, hyperéchogène par rapport au myomètre. L´échogénicité de l´endomètre augmente en cette période périovulatoire: il devient isoéchogène par rapport au myomètre. La ligne qui sépare l´endomètre du myomètre apparaît très nette et hyperéchogène. En marge de l´endomètre, la glaire cervicale est visible de façon fugace à cette période, sous forme d´une image liquidienne qui décolle le canal cervical.

Dans la période postovulatoire immédiate, on observe une modification rapide et importante de l´échostructure de l´endomètre: l´hyperéchogénicité de la ligne de démarcation entre l´endomètre et le myomètre se propage vers la lumière utérine centrale, ce qui confère un aspect hyperéchogène et flou à cette zone. Simultanément, la ligne centrale qui séparait les deux feuillets de l´endomètre s´épaissit, gagnant la profondeur de chaque feuillet. En quelques heures, immédiatement après l´ovulation, l´endomètre devient ainsi totalement hyperéchogène et la lumière utérine n´est plus visible. L´endomètre continue de s´épaissir pour atteindre 12 à 14 mm. Ces modifications sont synchrones avec le début de la phase lutéale.

En phase lutéale, deux variations physiologiques sont à connaître, concernant d´une part l´épaisseur de l´endomètre et d´autre part l´aspect hyperéchogène. L´épaisseur de l´endomètre est susceptible de varier selon l´âge de la femme comme l´ont démontré Fitzgerald *et al*. [4] qui observent une épaisseur maximale moyenne de l´endomètre lutéal de 12,1 mm chez des femmes de 21 à 25 ans et de 15,9 mm chez des femmes de 37 à 45 ans (p < 0,001). Quant à l´hyperéchogénicité habituellement très homogène en milieu de phase lutéale, elle peut être remplacée par un aspect inhomogène de l´endomètre, associé à une faible fertilité potentielle selon Check et al. [5].

On note une augmentation du flux vasculaire les jours précédant l'ovulation, suivie par un deuxième pic 3 jours plus tard. Ces variations démontrent qu'il existe une régulation spécifique pour préparer la réceptivité endométriale. Les vaisseaux en provenance de la zone jonctionnelle peuvent alors pénétrer dans l'endomètre jusqu'au niveau de la ligne cavitaire: ces aspects témoignent d'une réceptivité endométriale optimale. La zone jonctionnelle, qui est la portion de myomètre adjacent à l'endomètre (ou zone sub-endométriale du myomètre d'origine müllerienne). Ce sont plus particulièrement les atteintes de la zone jonctionnelle qui sont susceptibles de retentir sur la nidation. On l'identifie comme une zone hypoéchogène de 5 mm. Cette zone, qui n'était visualisable que par IRM, est aujourd'hui facilement identifiable lors l'échographie pelvienne, en particulier grâce à l'échographie 3D (phénomènes de sommation spatiale). Son épaisseur peut varier au cours de la vie génitale, pouvant s'épaissir après 35 ans ou s'atrophier en cas de contraception prolongée ou d'hypo-oestrogénie. Étiologie de l´infertilité d´origine endométriale détectable par échographie endovaginale: les lésions endométriales détectables par l´échographie sont les anomalies congénitales de l´utérus, les fibromes, les polypes, et les synéchies. La sensibilité globale de l´EEV dans la détection des anomalies endométriales chez les patientes infertiles a été évaluée à 98,9%, avec une valeur prédictive positive de 94,3% et une valeur prédictive négative de 5,5%. La spécificité globale d´un examen échographique normal est de 31,3%, avec une valeur prédictive négative de 71,4% [6].

**Figure 1:**
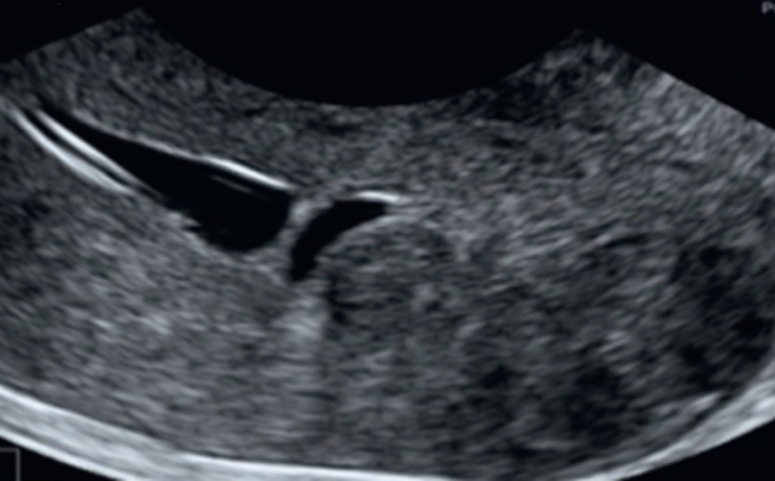
coupe sagittale d'un utérus avec une synéchie fundique

Les synéchies intra-utérines: elles surviennent à la suite d´infections, de traumatismes, de gestes opératoires endo-utérins, généralement dilatation et curetage en post-partum. Les synéchies sont des adhérences entre les parois opposées de la cavité utérine et peuvent être partielles ou complètes et peuvent concerner les différentes parties de l´utérus: corps utérin, isthme ou canal cervical. Le syndrome d´Asherman correspond à une cicatrice avec oblitération de la cavité endo-utérine par les synéchies provoquant une hypo ou une aménorrhée. Ce syndrome a été initialement décrit à la fin du 19e siècle alors que la tuberculose en était le facteur étiologique principal. Les synéchies sont à l´origine d´un défaut d´implantation de l´embryon, notamment quand elles touchent le fond utérin, elles peuvent également agir en amont de la fécondation, du fait de leur impact potentiel sur: Altération de la migration des spermatozoïdes; Altération vasculaire endométriale; Réduction de la taille de l´expansion de la cavité utérine. Les synéchies, accolements cavitaires antéropostérieurs ou marginaux, sont moins bien étudiables, parfois identifiables sous la forme de stops cavitaires. Les reconstructions 3D rendent plus sensible l'étude de la cavité, permettant une sommation d'images difficilement réalisable en 2D, où l'épaisseur de coupe n'est pas modulable par l'opérateur. L'accès à une vision verticale ou coronale de la cavité utérine permet en outre une meilleure localisation des lésions, offrant une vision anatomique voire hystéroscopique (Figure 1). Polypes endométriaux et léiomyomes.

Il existe encore moins de certitudes concernant l´impact des polypes sur la fertilité. Il n´existe que peu de travaux étudiant l´association entre polypes endométriaux et infertilité. La fréquence des polypes dans la population des femmes infertiles varie de 0 à 60% selon les études [7, 8]. Dans une série de FIV, il n´y avait pas de différence en termes de taux d´implantation et d´avortements entre un groupe de 33 patientes ayant des polypes endométriaux et un groupe de 280 patientes sans polypes [9]. Varasteih *et al*. [10] n´ont pu démontrer aucun bénéfice de la résection de polypes sur les taux de grossesse dans une étude de 23 patientes infertiles. Le mécanisme par lequel les polypes endométriaux peuvent retentir sur l´infertilité reste aussi mal compris et peut être lié à l´effet mécanique sur le transport spermatique ou sur l´implantation embryonnaire ou par l´effet de l´augmentation de la sécrétion de facteurs inhibiteurs, comme la glycodéline qui peut inhiber l´action des lymphocytes natural killer [11].

Les polypes endométriaux se présentent en échographie, sous forme de plages hyperéchogènes, intracavitaires, leur taille peut aller de quelques millimètres à 5 cm de diamètre [12]. L´EEV est très sensible pour la détection de polypes endométriaux, il est difficile de différencier les polypes endométriaux des léiomyomes sous muqueux ou d´un carcinome endométrial. L´échographie avec Doppler peut aider à différencier les polypes endométriaux des léiomyomes sous muqueux en montrant la présence d´une artère unique dans les polypes alors qu´il existe de nombreux vaisseaux dans les léiomyomes. Cependant, une artère nourricière unique peut aussi être détectée en cas de carcinome endométrial. Entre J3 et J5 du cycle, l'endomètre est fin, plutôt hyperéchogène en raison du flux menstruel et de son abrasion, il peut être difficile de mettre en évidence un polype muqueux. S'il est de petite taille, il se confond avec le matériel menstruel. Il est parfois suspecté par la mise en évidence d'un pédicule vasculaire au sein de l'endomètre. Tout doute sur la réalité de l'image doit amener à un contrôle en phase proliférative.

**Figure 2:**
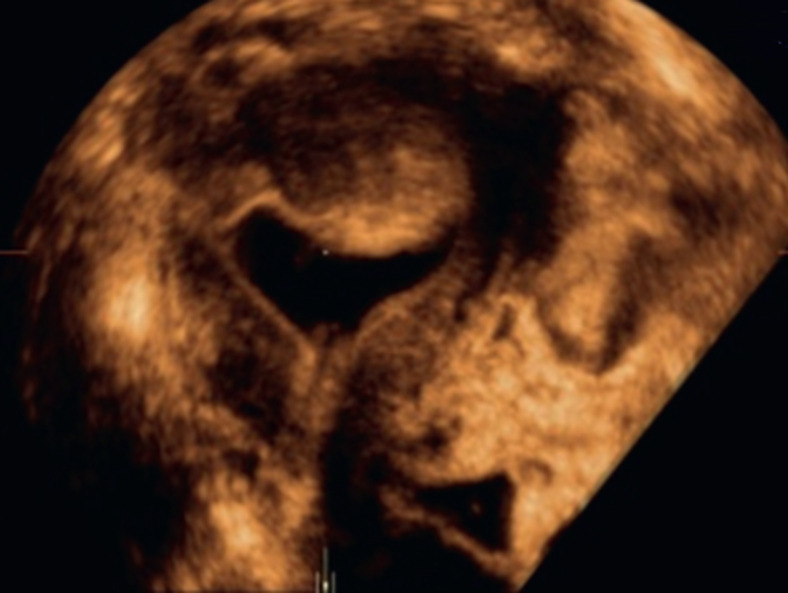
myome fundique latéralisé à gauche, transmural, avec composante intracavitaire: coupe coronale myome de grade II: moins de 50% de son volume est endocavitaire

L´HSO avec injection de sérum physiologique améliore également la détection de la pathologie endométriale [13]. Elle permet la détection de léiomyomes sous muqueux, pour déterminer leur taille, leur topographie et leur proportion intracavitaire. L'HSO permet de faire aisément le diagnostic différentiel entre une hypertrophie endométriale et un polype. En cas de fibrome, elle permet de bien préciser la localisation, le degré d'invasion intracavitaire, l'angle de raccordement avec la muqueuse adjacente et le mur postérieur de sécurité (au moins 5 mm) pour guider une éventuelle intervention chirurgicale (Figure 2).

### Fibromes sous-muqueux

Ils peuvent gêner la reproduction, soit en bouchant la trompe de Fallope ou le canal cervical, ou en gênant l´implantation. Cependant, ils sont rarement la seule cause d´infertilité [14, 15]. Les léiomyomes sont classés en fonction de leur topographie. Un myome sous-séreux constitue rarement une cause d'infertilité, mais un volumineux myome intramural ou, a fortiori, un myome sous muqueux peut constituer un obstacle éventuel à la nidation. La relation infertilité et fibrome utérins reste toujours controversée. Il est admis que les taux de grossesse en AMP sont plus bas en cas de présence de fibromes sous-muqueux ou interstitiels déformant la cavité utérine [7]. Approximativement, 5 à 10% des femmes infertiles ont au moins un fibrome et les fibromes utérins représentent l´unique cause de l´infertilité dans 1 à 2,4% des cas [16]. Il a été démontré dans une méta-analyse des études faites à partir de fécondation in vitro (FIV) que les fibromes sous-muqueux compromettent significativement le pronostic en AMP, alors que ni les fibromes sous-séreux ni interstitiels n´ont un effet néfaste sur la fertilité [17]. Plusieurs études suggèrent un effet néfaste des gros fibromes (> 4 cm) sur les taux de grossesse et les taux d´implantation en cycles de FIV [18, 19]. Cependant, d´autres études n´ont pas retrouvé de lien [20, 21].

L´échographie permet un dépistage rapide des léiomyomes. Les aspects échographiques comprennent une augmentation de volume de l´utérus, une déformation des contours et de la cavité utérine et une modification de l´échostructure. Les léiomyomes sont fréquemment hypoéchogènes mais leur aspect est variable. De petites lésions sont difficiles à détecter par échographie. Différence entre myomes intracavitaires et polypes: Quand un myome est intracavitaire (myome de grade 0 ou 1 de la FIGO), il est parfois difficile d'en faire la distinction avec un polype, d'autant que le polype peut apparaître iso-échogène au myomètre. Les aspects à rechercher pour faire le diagnostic différentiel à l'échographie sont présentés dans le Tableau 1.

**Tableau 1:** polype et myome sous-muqueux: diagnostic différentiel

	Polype	Myome sous-muqueux
**échostructure**	Plus souvent hyperéchogène	Plus souvent hypo-échogène
**Bridge edge liseré endométrial**	Présent	Absent
**Cône d´ombre Postérieur**	Absent	Présent
**Morphologie**	Ovoïdale	Arrondie
**Compressibilité**	Minime	Absente
**Doppler**	Pédicule vasculaire	Encorbellement Vasculaire

**Tableau 1:** polype et myome sous-muqueux: diagnostic différentiel

	Polype	Myome sous-muqueux
**échostructure**	Plus souvent hyperéchogène	Plus souvent hypo-échogène
**Bridge edge liseré endométrial**	Présent	Absent
**Cône d´ombre Postérieur**	Absent	Présent
**Morphologie**	Ovoïdale	Arrondie
**Compressibilité**	Minime	Absente
**Doppler**	Pédicule vasculaire	Encorbellement Vasculaire

### Les cloisons utérines

Le dépistage des malformations génitales et notamment des cloisons utérines fait partie des objectifs du bilan initial d´infertilité. L'utérus cloisonné est la plus fréquente des malformations d'origine müllerienne (55%). L'utérus bicorne monocervical est bien moins fréquent (10%). L'utérus bicorne bicervical est encore plus rare (5%). Le retentissement des cloisons utérines sur la reproduction est toujours sujet de controverses. Leur incidence chez les femmes en âge de procréation est estimée entre 0,5 et 6% [22]. La responsabilité des cloisons utérines en cas d´avortements à répétition ou d´accouchements prématurés est admise et il est consensuel de les opérer après de tels antécédents.

La responsabilité des cloisons utérines dans l´infertilité est non démontrée. Leur incidence chez les patientes infertiles est variable entre 16 et 24% selon les auteurs [22-24]. Cependant, chez les patientes connues infertiles, la présence d´une cloison utérine semble réduire les chances de grossesse et de naissance vivante [25]. La majorité des études de PMA (procréation médicalement assistée) montrent des taux de naissances vivantes plus bas en cas de présence de cloisons utérines non opérées, à cause de taux d´avortements et d´accouchements prématurés plus élevés mais ne montrent pas une baisse des taux d´implantation [26].

**Figure 3:**
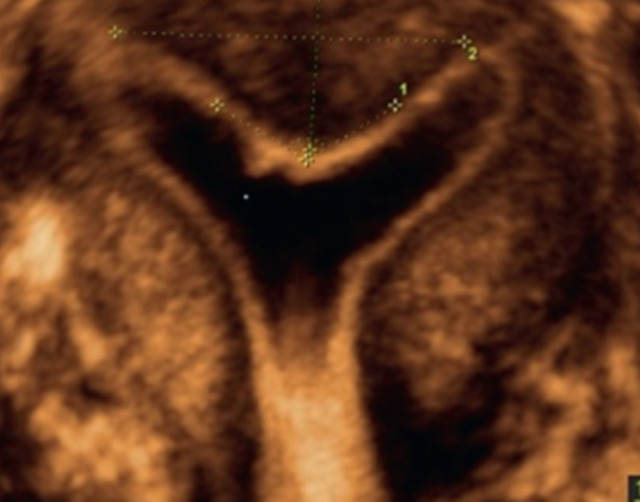
utérus avec septum: coupe coronale: on peut apprécier les dimensions du septum

Les critères diagnostiques les plus importants sont: la forme de la cavité et la morphologie de la séreuse et du col. La classification de Woelfer *et al*.de 2001 est pratique, car elle définit la différence entre utérus cloisonné et bicorne par la présence d'une incisure de la séreuse de hauteur égale ou inférieure à 10 mm sur la reconstruction 3D coronale (Figure 3). Le groupe de travail CONUTA ESHRE/ESGE a réalisé en 2013 une nouvelle classification en étudiant séparément l'utérus (U), le cervix (C) et le vagin (V) [27, 28]. Avec cette classification l'utérus cloisonné est diagnostiqué par la présence d'une incisure de la séreuse que ne dépasse pas 50% du myomètre fundique. Et au contraire, l'utérus sera bicorne s'il présente une incisure de la séreuse profonde représentant plus de 50% du myomètre fundique. L´EEV sera réalisé de préférence en deuxième partie de cycle, l´épaississement physiologique de l´endomètre favorise le diagnostic. L´échographie tridimensionnelle permet de réaliser un diagnostic plus précis, avec une très bonne sensibilité [29, 30].

## Conclusion

L´échographie est considérée l´une des plus grandes avancées technologiques dans l´imagerie gynécologique à cause de son caractère non invasif, peu couteux et de sa performance, en cas d´infertilité d´origine endométriale son rôle est variable selon les différentes étiologies. La possibilité de diagnostiquer des anomalies spécifiques de la cavité endométriale dépend directement de la phase du cycle menstruel au moment de l´examen. Les polypes endométriaux sont mieux vus en phase proliférative. Les léiomyomes sous muqueux et les synéchies sont mieux mis en évidence en phase sécrétoire. En outre, l´hystérosonographie a l´avantage de permettre l´évaluation de la cavité endométriale à n´importe quel moment du cycle. Cependant, en cas de pathologies endométriales minimes, l´échographie vaginale devient insuffisante pour déterminer la nature et la localisation des anomalies.
